# Association between the body mass index and outcomes of patients resuscitated from out-of-hospital cardiac arrest: a prospective multicentre registry study

**DOI:** 10.1186/s13049-021-00837-x

**Published:** 2021-01-28

**Authors:** Heekyung Lee, Jaehoon Oh, Hyunggoo Kang, Tae Ho Lim, Byuk Sung Ko, Hyuk Joong Choi, Seung Min Park, You Hwan Jo, Jong Suk Lee, Yoo Seok Park, Young-Hoon Yoon, Su Jin Kim, Young-Gi Min, for the Korean Cardiac Arrest Research Consortium (KoCARC) Investigators, Do Kyun Kim, Sang Kuk Han, Phil Cho Choi, Sang O. Park, Jong Won, Han Sung, Sung Hyuk Choi, Min Seob Sim, Gun Tak Lee, Shin Ahn, Jong Whan Shin, Sang Hyun Park, Keun Hong Park, In Cheol Park, Tae Young Kong, Kyoung Won Lee, Chu Hyun Kim, Youngsuk Cho, Gu Hyun Kang, Yong Soo Jang, Seok Ran Yeom, Sang Kyoon Han, Jae Hoon Lee, Jeong Bae Park, Hyun Wook Ryoo, Kyung Woo Lee, Tae Chang Jang, Jae-hyug Woo, Woon Jeong Lee, Seon Hee Woo, Sung Hyun Yun, Tae Jin Cho, Sun Pyo Kim, Yong Jin Park, Jin Woong Lee, Wonjoon Jeong, Sung Soo Park, Jae Kwang Lee, Ryeok Ahn, Wook Jin Choi, Young Gi Min, Eun Jung Park, Joong Hee Kim, In Byung Kim, Ki Ok Ahn, Han Jin Cho, Seung Cheol Lee, Sang Hun Lee, Young Sik Kim, Young Rock Ha, Jin Sik Park, Myoung Woo Lee, Dai Han Wi, Sang Ook Ha, Won Seok Yang, Ok Jun Kim, Tae Nyoung Chung, Soon Joo Wang, Hang A. Park, Jun Hwi Cho, Chan Woo Park, An Mu Eob, Tae Hun Lee, Sang Chul Kim, Hoon Kim, Han Joo Choi, Chan Young Koh, Jung Won Lee, Dong Wook Lee, Tae Oh. Jung, Jae Chol Yoon, Dai Hai Choi, Jung Tae Choi, Jin Hee Jeong, Soo Hoon Lee, Ji Ho Ryu, Maeng Real Park, Won Kim, Sung Wook Song, Woo Jung Kim, Joon-myoung Kwon, Eui Hyuk Kang, Sang Chan Jin, Tae-kwon Kim, Seong Chun Kim, Sung Oh. Hwang, Sang Do Shin, Hyuk Jun Yang, Sung Phil Chung, Sung Woo Lee, Kyung Jun Song, Seung Sik Hwang, Gyu Chong Cho, Sung Woo Moon, Kyoung Chul Cha, Won Young Kim, Sang Hoon Na, Young Ho Kwack, Joo Yeong Kim, Jeong Ho Park, Sun Young Lee, Jung Eun Kim

**Affiliations:** 1 Department of Emergency Medicine, College of Medicine, Hanyang University, 222 Wangsimni-ro, Seongdong-gu, Seoul, 04763 Republic of Korea; 2 Department of Emergency Medicine, Seoul National University Bundang Hospital, Gyeonggi-do, Republic of Korea; 3 Department of Emergency Medicine, College of Medicine, Kyung Hee University, Seoul, Republic of Korea; 4 Department of Emergency Medicine, Yonsei University College of Medicine, Seoul, Republic of Korea; 5 Department of Emergency Medicine, College of Medicine, Korea University, Seoul, Republic of Korea; 6 Department of Emergency Medicine, Ajou University School of Medicine, Suwon, Republic of Korea

**Keywords:** Obesity, Body mass index, Out-of-hospital cardiac arrest

## Abstract

**Background:**

The effects of the body mass index (BMI) on outcomes of patients resuscitated from cardiac arrest are controversial. Therefore, the current study investigated the association between the BMI and the favourable neurologic outcomes and survival to discharge of patients resuscitated from out-of-hospital cardiac arrest (OHCA).

**Methods:**

This multicentre, prospective, nationwide OHCA registry-based study was conducted using data from the Korean Cardiac Arrest Resuscitation Consortium (KoCARC). We enrolled hospitals willing to collect patient height and weight and included patients who survived to the hospital between October 2015 and June 2018. The included patients were categorised into the underweight (< 18.5 kg/m^2^), normal weight (≥18.5 to < 25 kg/m^2^), overweight (≥25 to < 30 kg/m^2^), and obese groups (≥30 kg/m^2^) according to the BMI per the World Health Organization (WHO) criteria. The primary outcome was a favourable neurologic outcome; the secondary outcome was survival to discharge. Univariate and multivariate analyses were performed to investigate the association between BMI and outcomes.

**Results:**

Nine hospitals were enrolled; finally, 605 patients were included in our analysis and categorised per the WHO BMI classification. Favourable neurologic outcomes were less frequent in the underweight BMI group than in the other groups (*p* = 0.002); survival to discharge was not significantly different among the BMI groups (*p* = 0.110). However, the BMI classification was not associated with favourable neurologic outcomes or survival to discharge after adjustment in the multivariate model.

**Conclusion:**

The BMI was not independently associated with favourable neurologic and survival outcomes of patients surviving from OHCA.

**Supplementary Information:**

The online version contains supplementary material available at 10.1186/s13049-021-00837-x.

## Introduction

Overweight and obesity are defined as abnormal or excessive fat accumulation that may impair health. In 2016, a total of 39% of adults aged > 18 years were overweight, and 13% were obese [1]. The number of obese patients continues to be high worldwide, exceeding 30 to 40% in most sex and age groups in the United States [2]. The body mass index (BMI) is a simple weight-to-height ratio that is used to classify overweight and obese adult patients. A high BMI is associated with hypertension, type II diabetes mellitus, dyslipidaemia, and major cardiovascular diseases—such as heart failure, coronary heart disease, arrhythmia, and sudden cardiac arrest—resulting in increased risks of both out-of-hospital cardiac arrest (OHCA) and in-hospital cardiac arrest (IHCA) [3–6].

A chest compression depth of 5–6 cm is recommended during cardiopulmonary resuscitation to ensure high-quality chest compressions. However, this chest compression depth is insufficient for obese patients who experience cardiac arrest because thorax changes and airway management—including bag-valve mask ventilation—are difficult in such patients considering the changes in their anatomy and distortions [7–11]. The survival and neurologic outcomes of obese patients or patients with a high BMI who experience cardiac arrest are lower than those of other patients [12]. However, several previous studies investigated whether being overweight was associated with better survival outcomes in critically ill patients, including the post-resuscitation state, and this phenomenon is called the obesity paradox [13–15]. A large, multicentre, prospective study with data from a national registry showed that the BMI groups had similar rates of survival, except underweight patients with IHCA caused by non-shockable rhythm [16]. A meta-analysis that evaluated the effect of BMI on survival post resuscitation showed that overweight patients had a favourable outcome considering both survival and neurologic outcomes, especially in patients who experienced IHCA and patients without therapeutic hypothermia [17].

To the best of our knowledge, data are limited, and the results are debatable regarding the effect of obesity on the survival and neurologic outcomes of patients who experienced OHCA. Therefore, by using data from a nationwide multicentre registry, the current study aimed to assess the association between the outcomes and BMI in the four BMI groups after resuscitation in patients who experienced OHCA. We hypothesised that the neurologic outcomes after survival from the time of admission in patients who experienced OHCA would differ according to the BMI.

## Methods

### Study design and setting

We conducted the current multicentre prospective observational study using data from the Korean Cardiac Arrest Resuscitation Consortium (KoCARC) registry, which is a nationwide OHCA registry based on the Utstein templates and a hospital-based collaborative research network. The KoCARC registry included patients with OHCA transported to the participating emergency departments via emergency medical services with resuscitation efforts and who had a medical aetiology identified by emergency physicians. The registry excluded patients with a terminal illness documented in medical records, patients under hospice care, pregnant patients, and patients with a previously documented ‘Do Not Resuscitate’ card. Patients with cardiac arrest due to definite non-medical aetiology were also excluded, including trauma, drowning, poisoning, burn, asphyxia, or hanging. The data were collected via a standardised registry form and uploaded into a web-based electronic database registry; the quality of this registry is controlled by the quality management committee [18, 19]. The project was registered at ClinicalTrials.gov (identifier NCT03222999) and ethically reviewed and approved by the institutional review board (IRB) of the 62 participating hospitals. The study was waived for informed consent by the IRB.

### Study population

The KoCARC registry consists of core variables and supplemental variables. The participating hospital can decide to investigate each of the supplemental variables, including height and weight. Among the participating hospitals, we enrolled hospitals willing to include height and weight to minimise potential risk for bias. We included patients who survived the trip to the hospital—for admission to the enrolled hospital from the KoCARC registry—between October 2015 and June 2018. We excluded patients who were aged < 18 years, those who were transferred from other hospitals to the enrolled hospital, and those without any information about weight, height, or the Cerebral Performance Category (CPC). Additionally, we compared the baseline characteristics between the whole cohort of the registry and enrolled patients.

### Data extraction and definition

Information about the KoCARC database, data elements, and quality assurance has been previously published [19]. We extracted the following KoCARC core data and supplementary variables: 1) Patient domain: age, sex, witnessed arrest, arrest location, bystander cardiopulmonary resuscitation (CPR), first monitored rhythm, comorbidities (hypertension, diabetes, and dyslipidaemia). 2) Pre-return of spontaneous circulation (ROSC) process domain: response times (no-flow time was defined as the time between the cardiac arrest and the initiation of CPR by a medical provider; low flow time was defined as the time between active CPR by a bystander and/or a medical provider and ROSC; it is equal to the total arrest time minus the no-flow time), pre-hospital ROSC was defined as the restoration of a palpable pulse before arrival at the hospital, pre-hospital adrenaline use, pre-hospital airway control type (bag-valve mask, supraglottic airway, or endotracheal intubation). 3) Post-resuscitation process domain: mental state at admission (alert, drowsy to stuporous, or coma), reperfusion coronary angiography (CAG), CAG and percutaneous coronary intervention (PCI), tissue plasminogen activator (tPA), targeted temperature management (TTM), pH, lactate, glucose, first systolic/diastolic blood pressure, and pulse rate after ROSC. The enrolled patients were classified into the underweight (< 18.5 kg/m^2^), normal weight (≥18.5 to < 25 kg/m^2^), overweight (≥25 to < 30 kg/m^2^), and obese groups (≥30 kg/m^2^) according to the BMI per the World Health Organization (WHO) criteria.

### Outcome variables and subgroup analysis

The primary outcome was a favourable neurologic outcome defined as CPC of 1 or 2 at the time of hospital discharge. Patients had a CPC of 1 if they had good cerebral performance and were conscious, alert, and able to work with a possible mild neurologic or psychological deficit. Patients had a CPC of 2 if they had a moderate cerebral disability and were conscious, had sufficient cerebral function for independent activities of daily life, and were able to work in sheltered environments. This performance scale indicates mortality as a CPC of 5, defined as death or brain death [20]. The secondary outcome was survival to discharge, and we investigated factors that affected mortality. Moreover, we classified patients into the TTM and non-TTM groups and compared the favourable neurologic outcome and survival discharge according to the BMI in each group after adjusting the confounders.

### Statistical analysis

The KoCARC registry compiled and released data with a standard spreadsheet application (Excel 2016; Microsoft, Redmond, USA). Continuous baseline variables were presented as the median and interquartile range and analysed using the Shapiro-Wilk test for normality distribution. The Kruskal-Wallis test was used to compare groups with unsatisfied to normally distributed continuous variables. Categorical variables were presented as the number and percentages and analysed by using a chi-squared test. Two-tailed *p*-values < 0.05 indicated a significant difference. The logistic regression method was used for multivariate analysis to determine the association between the BMI and CPC at discharge and the survival discharge rate, independent of the confounders. The Hosmer-Lemeshow test was used to confirm the logistic model calibrations. Subgroup analysis was performed using forest plots, and the p-values were calculated for determining the effect of TTM on the association between the BMI and outcomes. SAS 9.4 (SAS Institute Inc., Cary, USA) and MedCalc version 17.2 (MedCalc Software, Ostend, Belgium) were used for all analyses.

## Results

**Fig. 1:**
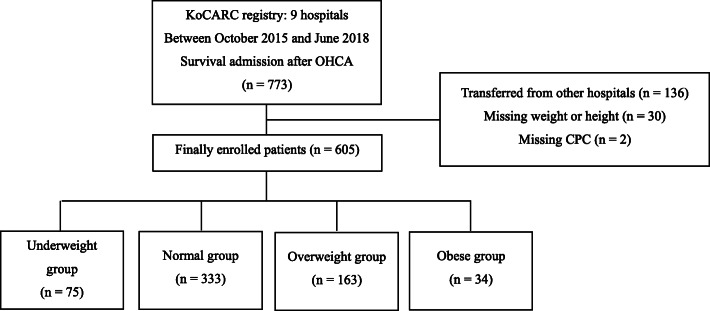
Flow chart of the study. KoCARC, Korean Cardiac Arrest Resuscitation Consortium; OHCA, out-of-hospital cardiac arrest; CPC, Cerebral Performance Category

Among 33 hospitals, 9 hospitals entered the weight and height of the registered patients. A total of 773 patients who survived to admission after OHCA were eligible; 168 patients were excluded according to the exclusion criteria: 136 were transferred from other hospitals, and 32 had missing data for height, weight, or CPC. Finally, 605 patients were included in our analysis and categorised per the WHO BMI classification (Fig. 1).

The baseline characteristics of all the included patients and those in each BMI group are summarised in Table 1 and the comparison with the whole registry in Supplemental Table 1. There were significant differences among the four groups in age, first monitored rhythm by emergency medical services, performance of TTM, pre-hospital ROSC, reperfusion attempt, and first serum lactate and glucose levels. Patients in the underweight group (*n* = 75, 12.4%) were relatively older and did not regularly show shockable rhythm in the first monitored rhythm when compared to those in the other groups. Moreover, patients in the underweight group experienced pre-hospital ROSC and treatment with TTM and reperfusion therapy with lower frequency than those in the other groups did. Furthermore, the first checked serum glucose levels were lower, and the lactate levels were higher in the underweight group than in the other groups. Other variables—including sex, witness arrest, bystander CPR, comorbidities, duration of cardiac arrest, and mentation at admission—were not significantly different among the groups.

**Table 1:** Baseline characteristics stratified by body mass index

Variables	Total (*n* = 605)	Underweight group (*n* = 75)	Normal group (*n* = 333)	Overweight group (*n* = 163)	Obese group (*n* = 34)	*p*-value*
Patient domain						
Age, years, median (IQR)	63 (53–74)	71 (62–79)	63 (54–75)	61 (51–72)	56.5 (42–72)	<.001
Sex, male (%)	422 (69.75)	53 (70.67)	233 (69.97)	115 (70.55)	21 (61.76)	0.774
Witnessed arrest (%)	449 (74.21)	55 (73.33)	244 (73.27)	125 (76.69)	25 (73.53)	0.870
Arrest location (*n* = 601)						
Home/residence (%)	320 (53.24)	48 (64)	172 (52.12)	85 (52.47)	15 (44.12)	0.185
Other (%)	281 (46.76)	27 (36)	158 (47.88)	77 (47.53)	19 (55.88)	
Bystander response (*n* = 602)						
CPR by bystander (%)	307 (51)	39 (52.7)	164 (49.4)	84 (51.53)	20 (60.61)	0.645
No CPR by bystander (%)	295 (49)	35 (47.3)	168 (50.6)	79 (48.47)	13 (39.39)	
First monitored rhythm (*n* = 597)						
Shockable rhythm (%)	250 (41.88)	11 (14.86)	149 (45.29)	72 (45)	18 (52.94)	<.001
Non-shockable rhythm (%)	347 (58.12)	63 (85.14)	180 (54.71)	88 (55)	16 (47.06)	
Comorbidities (*n* = 603)						
Hypertension (%)	294 (48.76)	31 (41.33)	153 (46.08)	93 (57.41)	17 (50)	0.058
Diabetes (%)	190 (31.51)	16 (21.33)	102 (30.72)	60 (37.04)	12 (35.29)	0.102
Dyslipidaemia (%)	40 (6.63)	2 (2.67)	22 (6.63)	12 (7.41)	4 (11.76)	0.320
Pre-ROSC process domain						
No flow time, minutes, median (IQR)	7 (5–10)	6 (3–10)	7 (5–11)	7 (5–10)	6 (5–10)	0.446
Low flow time, minutes, median (IQR) (*n* = 604)	25 (12–40)	26 (18–38)	24.5 (11–39)	25 (11–42)	24.5 (15–54)	0.655
Pre-hospital ROSC (%)	206 (34.05)	15 (20)	129 (38.74)	51 (31.29)	11 (32.35)	0.015
Pre-hospital adrenaline use (%)	62 (10.25)	5 (6.67)	39 (11.71)	16 (9.82)	2 (5.88)	0.465
Pre-hospital airway						
Bag-valve mask (%)	234 (38.68)	34 (45.33)	120 (36.04)	66 (40.49)	14 (41.18)	0.427
Supraglottic airway (%)	300 (49.59)	29 (38.67)	173 (51.95)	82 (50.31)	16 (47.06)	
Endotracheal intubation (%)	71 (11.74)	12 (16)	40 (12.01)	15 (9.2)	4 (11.76)	
Post-ROSC process domain						
Mental state at admission						
Alert (%)	60 (9.92)	4 (5.33)	33 (9.91)	20 (12.27)	3 (8.82)	0.333
Drowsy to stuporous (%)	63 (10.41)	6 (8)	30 (9.01)	22 (13.5)	5 (14.71)	
Coma (%)	482 (79.67)	65 (86.67)	270 (81.08)	121 (74.23)	26 (76.47)	
Reperfusion attempted						
None (%)	297 (49.09)	52 (69.33)	164 (49.25)	68 (41.72)	13 (38.24)	0.002
CAG only (%)	170 (28.1)	10 (13.33)	93 (27.93)	54 (33.13)	13 (38.24)	
CAG and PCI (%)	96 (15.87)	6 (8)	60 (18.02)	26 (15.95)	4 (11.76)	
tPA (%)	42 (6.94)	7 (9.33)	16 (4.8)	15 (9.2)	4 (11.76)	
TTM (%)	225 (37.19)	17 (22.67)	122 (36.64)	72 (44.17)	14 (41.18)	0.015
pH, median (IQR) (*n* = 600)	7.07 (6.92–7.25)	7.06 (6.95–7.27)	7.11 (6.91–7.27)	7.03 (6.9–7.22)	7.11 (6.98–7.28)	0.212
Lactate, mmol/l, median (IQR) (*n* = 593)	10.6 (7.6–14)	11.7 (9–14.39)	10.285 (7.2–13.2)	11 (8.2–15.29)	9.4 (6.5–13.2)	0.008
Glucose, mg/dl, median (IQR) (n = 603)	265 (193–338)	229 (146–313)	268 (207–335)	265 (190–378)	268.5 (228–362)	0.039
First systolic BP after ROSC, mmHg, median (IQR) (*n* = 590)	122.5 (92–152)	130 (80–149)	120 (90–149)	124.5 (95.5–160)	121 (98–156)	0.482
First diastolic BP after ROSC, mmHg, median (IQR) (*n* = 589)	75 (59–93)	79 (56–93)	74 (56–91)	77 (60–96)	72 (57–90)	0.428
First pulse rate after ROSC, beats/min, median (IQR) (*n* = 586)	100 (81–123)	99 (75–132)	96 (79–120)	108 (87–124)	102 (78–116)	0.062

*Abbreviations*: *IQR* interquartile range, *CAG* coronary angiography, *CPR* cardiopulmonary resuscitation, *PCI* percutaneous coronary intervention, *ROSC* return of spontaneous circulation, *tPA* tissue plasminogen activator, *TTM* targeted temperature management

Continuous variables are presented as the median (Q1, Q3) and tested by using the Kruskal-Wallis test, and categorical variables are presented as N (%) and tested by using the chi-squared test

*p < 0.05 was significant

The primary and secondary outcomes, according to the BMI classification, are summarised in Table 2. A favourable neurologic outcome was observed less frequently in the underweight group than in the other groups (*p* = 0.002), and survival to discharge was not significantly different among the groups (*p* = 0.110).

**Table 2:** Primary and secondary outcomes of groups according to body mass index classification on univariate analysis

Outcomes	Total (*n* = 605)	Underweight group (*n* = 75)	Normal group (*n* = 333)	Overweight group (*n* = 163)	Obese group (*n* = 34)	*p*-value
Favourable neurologic outcomes at hospital discharge	202 (33.39)	11 (14.67)	120 (36.04)	56 (34.36)	15 (44.12)	0.002
Survival to discharge	303 (50.08)	28 (37.33)	176 (52.85)	81 (49.69)	18 (52.94)	0.111

All variables are presented as the N (%) and tested by using the chi-squared test

*p < 0.05 was significant

Univariate and multivariate analyses of factors associated with favourable neurologic outcomes were performed to adjust confounders that could affect the primary outcome (Table 3). On univariate analysis, the underweight group was less frequently (odds ratio [OR], 0.305; 95% confidence interval [CI], 0.155–0.601, *p* = 0.003) and the obese group was more frequently (OR, 1.401; 95% CI, 0.687–2.859, *p* = 0.040) associated with favourable neurologic outcomes and survival to discharge than the normal group. Significant differences were observed in age, first monitored rhythm, low flow time, pre-hospital ROSC, mentation at admission, and reperfusion therapy on both univariate and multivariate analyses. However, the BMI was not associated with favourable neurologic outcomes after adjusting for confounders on multivariate analysis.

**Table 3:** Univariate and multivariate analyses of factors affecting favourable neurologic outcomes

Variables	Univariate analysis			Multivariate analysis (*N* = 559)		
	Unadjusted OR	95% CI for the OR	*p*-value*	Adjusted OR	95% CI for the OR	*p*-value*
Age, (per year)	0.946	0.933–0.959	<.001	0.927	0.901–0.953	<.001
Sex, (Female/male)	0.573	0.388–0.844	0.005	1.307	0.617–2.766	0.484
Witnessed arrest (Yes/No)	1.503	1.004–2.249	0.045	0.961	0.461–2.003	0.916
Arrest location (Home/Others)	0.476	0.337–0.672	<.001	0.703	0.366–1.35	0.290
Bystander response (CPR performed/CPR not performed)	2.069	1.463–2.927	<.001	0.658	0.325–1.332	0.245
First monitored rhythm (Shockable/Non-shockable)	14.456	9.456–22.101	<.001	5.869	2.646–13.018	<.001
Hypertension (Present/Absent)	0.573	0.406–0.808	0.002	1.193	0.579–2.459	0.632
Diabetes (Present/Absent)	0.449	0.302–0.667	<.001	0.547	0.252–1.188	0.127
Dyslipidaemia (Present/Absent)	1.685	0.882–3.22	0.114			
No flow time, (per minute)	0.992	0.97–1.014	0.470			
Low flow time, (per minute)	0.907	0.891–0.923	<.001	0.962	0.938–0.987	0.003
Pre-hospital ROSC (Observed/Not observed)	19.647	12.791–30.18	<.001	3.762	1.442–9.813	0.007
Pre-hospital adrenaline (Used/Not used)	0.446	0.232–0.858	0.016	1.103	0.319–3.814	0.877
Pre-hospital airway						
Bag-valve mask	Reference	–	–			
Supraglottic airway	0.769	0.537–1.101	0.152			
Endotracheal intubation	0.606	0.337–1.092	0.095			
Mental state at admission						
Alert	Reference	–	–	Reference	–	–
Drowsy to stuporous	0.357	0.136–0.938	0.037	3.924	0.81–19.022	0.006
Coma	0.036	0.016–0.081	<.001	0.226	0.065–0.782	<.001
Reperfusion attempted						
None	Reference	–	–	Reference	–	–
CAG only	9.106	5.818–14.252	<.001	2.636	1.193–5.823	0.057
CAG and PCI	7.482	4.457–12.561	<.001	5.027	1.869–13.521	0.001
tPA	0.821	0.305–2.207	0.696	0.353	0.069–1.802	0.021
TTM (Performed/Not performed)	0.934	0.658–1.327	0.705			
pH	632.387	193.43–999.999	<.001	1.706	0.196–14.837	0.628
Lactate (per mmol/l)	0.858	0.821–0.897	<.001	0.972	0.893–1.058	0.513
Glucose (per mg/dl)	0.998	0.996–0.999	0.008	0.998	0.994–1.001	0.136
First systolic BP after ROSC, (per mmHg)	1.007	1.003–1.011	0.003	1.011	0.996–1.025	0.145
First diastolic BP after ROSC, (per mmHg)	1.024	1.017–1.032	<.001	1.013	0.991–1.036	0.253
First pulse rate after ROSC, (per beat/min)	1.000	0.994–1.005	0.993			
BMI, kg/m^2^						
18.5 to 24.9	Reference	–	–	Reference	–	–
< 18.5	0.305	0.155–0.601	0.003	0.656	0.185–2.325	0.358
25.0 to 29.9	0.929	0.627–1.376	0.366	0.658	0.301–1.441	0.187
> 30.0	1.401	0.687–2.859	0.040	2.722	0.65–11.401	0.087

*Abbreviations*: *BMI* body mass index, *CAG* coronary angiography, *CI* confidence interval, *CPR* cardiopulmonary resuscitation, *OR* odds ratio, *PCI* percutaneous coronary intervention, *ROSC* return of spontaneous circulation, *tPA* tissue plasminogen activator, *TTM* targeted temperature management

*p < 0.05 was significant

Survival to discharge was associated with age, first monitored rhythm, diabetes, low flow time, pre-hospital ROSC, and mentation at admission on multivariate analysis. The underweight group had a lower rate of survival to discharge than the normal BMI group on univariate analysis (OR, 0.531; 95% CI, 0.318–0.889, *p* = 0.030). However, underweight, overweight, and obese BMI were not risk factors for survival to discharge after adjusting for influencing factors on multivariate analysis by using a logistic regression model (Table 4).

**Table 4:** Univariate and multivariate analyses of factors affecting survival to discharge

Variables	Univariate analysis			Multivariate analysis (N = 559)		
	Unadjusted OR	95% CI for the OR	*p*-value*	Adjusted OR	95% CI for the OR	*p*-value*
Age, (per year)	0.958	0.947–0.97	<.001	0.971	0.953–0.989	0.001
Sex, (Female/male)	0.61	0.43–0.867	0.006	0.789	0.478–1.302	0.354
Witnessed arrest (Yes/No)	1.695	1.171–2.453	0.005	1.434	0.849–2.422	0.178
Arrest location (Home/Others)	0.513	0.371–0.71	<.001	0.783	0.491–1.249	0.305
Bystander response (CPR performed/CPR not performed)	1.707	1.236–2.356	0.001	1.194	0.741–1.923	0.467
First monitored rhythm (Shockable/Non-shockable)	6.098	4.244–8.761	<.001	2.762	1.505–5.068	0.001
Hypertension (Present/Absent)	0.64	0.464–0.883	0.007	1.073	0.64–1.799	0.790
Diabetes (Present/Absent)	0.409	0.286–0.583	<.001	0.381	0.221–0.657	0.001
Dyslipidaemia (Present/Absent)	1.101	0.579–2.093	0.769			
No flow time, (per minute)	0.984	0.964–1.005	0.142			
Low flow time, (per minute)	0.939	0.927–0.95	<.001	0.969	0.953–0.986	<.001
Pre-hospital ROSC (Observed/Not observed)	10.845	7.075–16.624	<.001	2.161	1.011–4.619	0.047
Pre-hospital adrenaline (Used/Not used)	0.474	0.273–0.824	0.008	0.639	0.283–1.443	0.281
Pre-hospital airway						
Bag-valve mask	Reference	–	–			
Supraglottic airway	0.802	0.569–1.129	0.206			
Endotracheal intubation	0.703	0.413–1.199	0.196			
Mental state at admission						
Alert	Reference	–	–	Reference	–	–
Drowsy to stuporous	0.43	0.14–1.321	0.141	1.685	0.433–6.559	0.067
Coma	0.062	0.025–0.158	<.001	0.451	0.146–1.397	0.005
Reperfusion attempted						
None	Reference	–	–	Reference	–	–
CAG only	5.003	3.307–7.567	<.001	1.58	0.84–2.973	0.197
CAG and PCI	2.832	1.764–4.548	<.001	0.923	0.438–1.945	0.385
tPA	1.262	0.652–2.443	0.490	1.293	0.555–3.013	0.765
TTM (Performed/Not performed)	1.34	0.962–1.865	0.083			
pH	89.557	34.575–231.976	<.001	1.572	0.375–6.59	0.536
Lactate (per mmol/l)	0.868	0.835–0.903	<.001	0.958	0.907–1.011	0.115
Glucose (per mg/dl)	0.998	0.997–0.999	0.002	1	0.998–1.002	0.985
First systolic BP after ROSC, (per mmHg)	1.008	1.004–1.012	<.001	1.009	1–1.018	0.054
First diastolic BP after ROSC, (per mmHg)	1.021	1.014–1.028	<.001	0.999	0.983–1.015	0.931
First pulse rate after ROSC, (per beat/min)	1.003	0.997–1.008	0.340			
BMI, kg/m^2^						
18.5 to 24.9	Reference	–	–	Reference	–	–
< 18.5	0.531	0.318–0.889	0.030	0.854	0.425–1.719	0.889
25.0 to 29.9	0.881	0.606–1.282	0.697	0.856	0.49–1.495	0.861
> 30.0	1.004	0.495–2.035	0.474	0.621	0.211–1.823	0.496

*Abbreviations*: *BMI* body mass index, *CAG* coronary angiography, *CI* confidence interval, *CPR* cardiopulmonary resuscitation, *OR* odds ratio, *PCI* percutaneous coronary intervention, *ROSC* return of spontaneous circulation, *tPA* tissue plasminogen activator, *TTM* targeted temperature management

*p < 0.05 was significant

**Fig. 2:**
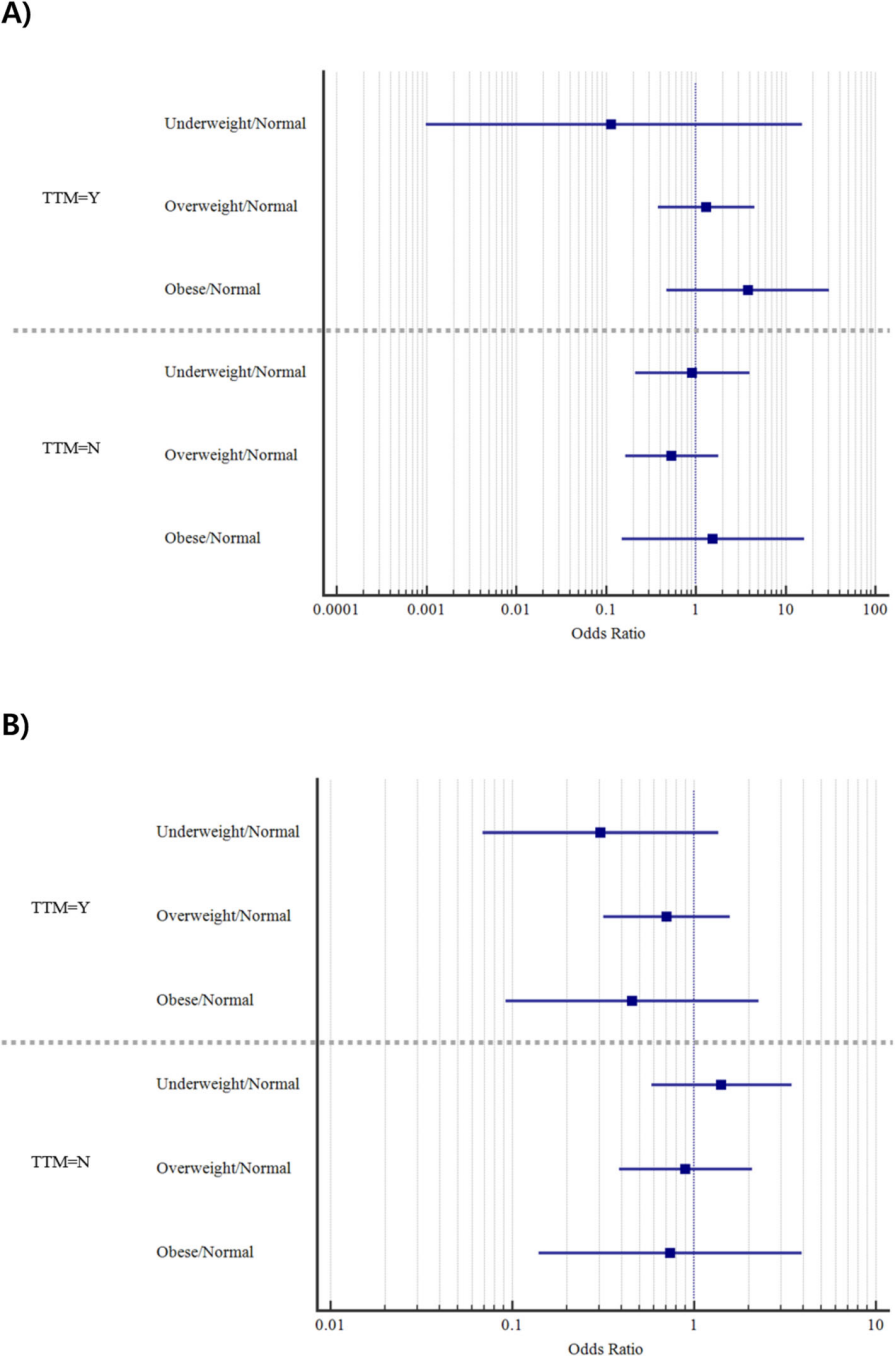
Comparison of the odds ratios between the body mass index groups for (**a**) favourable neurologic outcomes (*p* = 0.429) and (**b**) survival to discharge (*p* = 0.295) according to whether targeted temperature management was performed

On subgroup analysis, a low or high BMI was not a risk factor for poor neurologic outcomes or in-hospital mortality in both the TTM and non-TTM groups on multivariate analysis (Fig. 2).

## Discussion

In the current study, we aimed to investigate the relationship between the BMI and neurologic and survival outcomes of patients successfully resuscitated from OHCA. We found there were no significant differences in the survival outcomes among the four BMI groups. A favourable neurologic outcome was higher in the obese group and lower in the underweight group on univariate analysis. However, no differences were found after adjusting the confounding factors.

In previous studies and meta-analyses, there were no unified results about the association between the BMI and outcomes after cardiac arrest. A recent meta-analysis showed that the hospital survival rate was lower in the underweight group and higher in the overweight group, but no difference was found on a subgroup analysis of patients who experienced OHCA only [21]. Moreover, another meta-analysis found that overweight patients had better survival and neurologic outcomes after cardiac arrest, and it was amplified in the IHCA with TTM group [17]. However, these meta-analyses did not reflect confounding factors, and the result was different according to the included studies.

A multicentre prospective study showed that patients with a BMI > 30 kg/m^2^ had higher 30-day mortality among patients resuscitated from OHCA and managed with TTM [22]. In the present study, no differences were found in both neurologic and survival outcomes when the obese and normal groups managed with TTM were compared. Bunch et al. reported that the discharge rates and favourable neurologic outcomes were similar between the BMI groups among patients with ventricular fibrillation during OHCA [23]. They did not analyse the underweight group separately and only included a relatively small number of variables. The results of the current study showed that the underweight group had lower favourable neurologic outcomes and lower survival discharge rates than the normal weight group did on univariate analysis. However, there were no differences after adjusting the confounding factors. In line with the results of the current study, Testori et al. reported no relationship between the BMI and 6-month survival rate [14]. However, the overweight group had better neurologic outcomes in cardiac arrest survivors because Testori et al. included both OHCA and IHCA and adjusted fewer factors such as pre-hospital factors, age, and comorbidities. In the present study, we included only OHCA and adjusted more factors, including the mentation state, reperfusion attempt, and laboratory findings.

According to the results of a large, multicentre, prospective registry-based study about BMI and survival after IHCA, the survival rates were affected by shockable/non-shockable rhythm and BMI [16]. However, OHCA and IHCA have different baseline characteristics, and underweight patients may have poor nutritional and functional status or severe comorbidities in IHCA, and this is more prominent in IHCA than in OHCA [24].

These various results may be obtained owing to differences in the study population or study design. However, no study investigated the association between the BMI categories and neurologic or survival outcomes of patients surviving after OHCA treated with or without TTM. To the best of our knowledge, the current study is the first to compare the neurologic outcomes between the BMI groups resuscitated after OHCA by using a nationwide multicentre registry including shockable/non-shockable rhythms and the TTM/non-TTM groups.

Obesity is associated with a higher risk of cardiovascular disease, and high-quality CPR is difficult in obese patients [12, 25]. However, overweight and obese patients were relatively younger and frequently showed shockable rhythm [16, 23, 26]. Accordingly, overweight and obese patients more frequently experience ROSC before hospital arrival and are managed with TTM or CAG. The result of the present study showed a similar trend observed in past studies [14, 22]. Obesity seemed to be associated with a good prognosis on univariate analysis. However, other variables associated with obesity—such as age, first monitored shockable rhythm, and pre-hospital ROSC—were more independently associated with favourable neurologic or survival outcomes than BMI was. Obesity was not independently associated with outcomes after adjusting for these factors. Based on these results, high BMI patients are more likely to suffer arrest with shockable rhythms, hence more likely to have ROSC, live to reach the hospital, and receive TTM.

The current study has several limitations. First, although the registry-based study was prospective, the aim to investigate the association between the BMI and outcomes of ROSC in patients who experienced OHCA was not decided prior to the initiation of the KoCARC registry. Second, because weight and height were not core variables of the KoCARC registry, we could not enrol all participated hospitals. However, there were no differences in the important variables of baseline characteristics between the whole registry and the study cohort. Third, although the registry collected various data based on the Utstein-style templates, and we tried to analyse the maximum possible number of important factors that that could affect outcomes, there may be hidden confounders that could affect outcomes. Fourth, the height or weight might not have been accurately measured, and weight changes during the hospital stay were not investigated in the current study. Finally, the long-term outcomes were unknown. The KoCARC registry collected 6-month data for survival and the neurologic outcome. However, these data were not sufficient because the observation period was too short at the time of data analysis. Therefore, further studies are needed to investigate the association between the BMI and long-term neurologic and survival outcomes after OHCA.

## Conclusions

In this study, we found that BMI is not independently associated with favourable neurologic and survival outcomes in patients surviving after OHCA when the confounding factors were adjusted.

## Supplementary Information

**Additional file 1: Figure S1.** Proportion of each CPC categories by body mass index classification, A) Total enrolled patients B) patients managed with targeted temperature management C) patients not managed with targeted temperature management.

**Additional file 2: Table S1.** Comparison of baseline characteristics between patients of KoCARC registry and the study cohort.

## Data Availability

The datasets used and analyzed during the current study are available from the corresponding author on reasonable request.

## Abbreviations

BMI: Body mass index
OHCA: Out-of-hospital cardiac arrest
IHCA: In-hospital cardiac arrest
KoCARC: Korean Cardiac Arrest Resuscitation Consortium
CPC: Cerebral Performance Category
ROSC: Return of spontaneous circulation
CPR: Cardiopulmonary resuscitation
CAG: Coronary angiography
PCI: Percutaneous coronary intervention
tPA: tissue plasminogen activator
TTM: Targeted temperature management
WHO: World Health Organization
OR: Odds ratio
CI: Confidence interval
