# Supplementary figures and images for: Association between the body mass index and outcomes of patients resuscitated from out-of-hospital cardiac arrest: a prospective multicentre registry study
Source: Scand J Trauma Resusc Emerg Med. 2021 Jan 28;29:24. doi: 10.1186/s13049-021-00837-x (PMC7842019; doi:10.1186/s13049-021-00837-x)

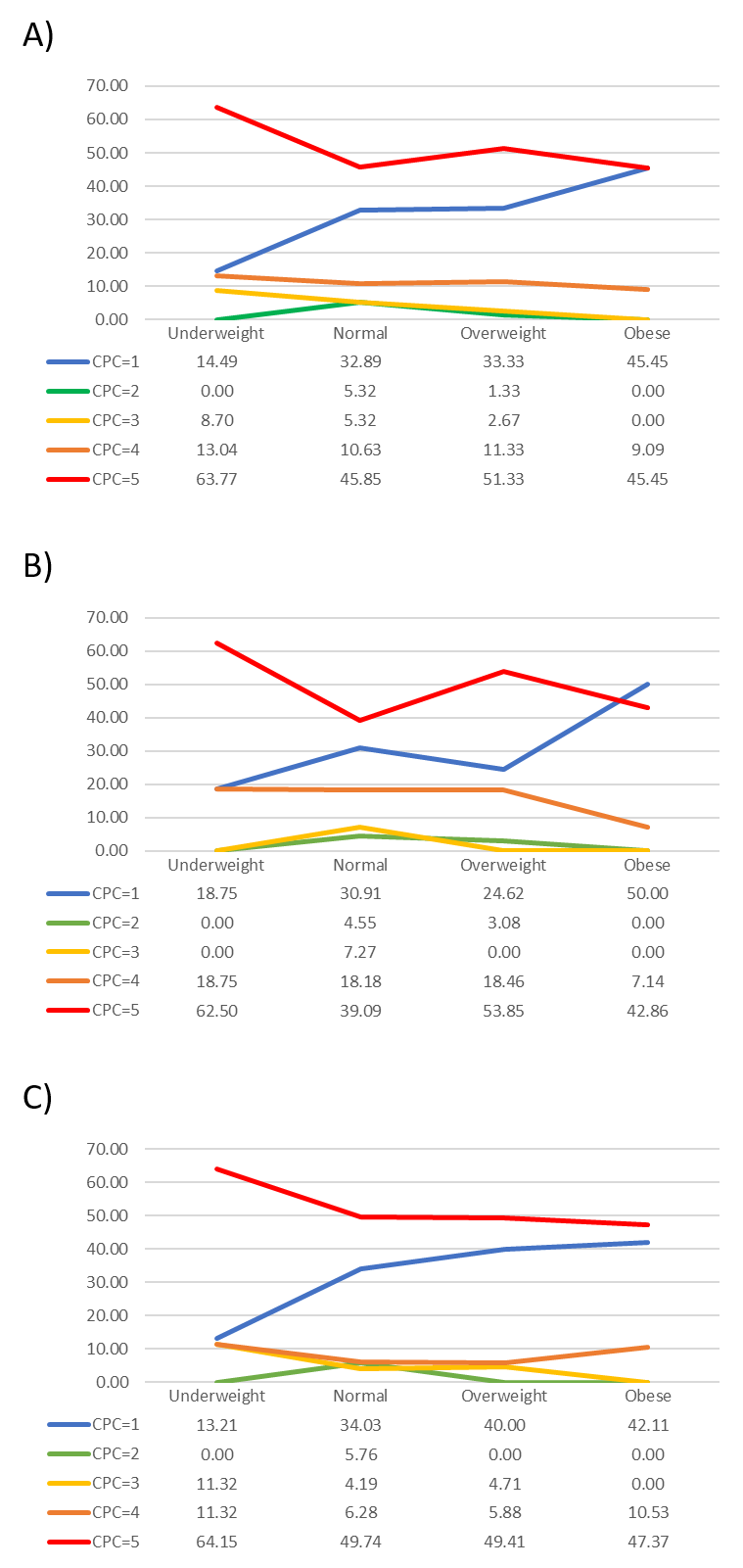

Supplement: Supplementary file 1 — Additional file 1: Figure S1. Proportion of each CPC categories by body mass index classification, A) Total enrolled patients B) patients managed with targeted temperature management C) patients not managed with targeted temperature management. [file 13049_2021_837_MOESM1_ESM.tif]
