# Supplementary material for: Association between the body mass index and outcomes of patients resuscitated from out-of-hospital cardiac arrest: a prospective multicentre registry study
Source: Scand J Trauma Resusc Emerg Med. 2021 Jan 28;29:24. doi: 10.1186/s13049-021-00837-x (PMC7842019; doi:10.1186/s13049-021-00837-x)
Supplement: Supplementary file 2 — Additional file 2: Table S1. Comparison of baseline characteristics between patients of KoCARC registry and the study cohort. [file 13049_2021_837_MOESM2_ESM.docx]

Supplemental table 1. Comparison of baseline characteristics between patients of KoCARC registry and the study cohort.

| Characteristics | KoCARC registry^a^ (n = 1743) | The study cohort (n = 605) | *p*-value* |
| --- | --- | --- | --- |
| Age, year | 62 (51-73) | 63 (53-74) | 0.075 |
| Gender, male | 1262 (72.4) | 422 (69.8) | 0.212 |
| First monitored rhythm, shockable (n = 2219) | 685 (42.4) | 250 (41.9) | 0.635 |
| TTM, performed (n = 2223) | 530 (32.8) | 225 (37.2) | 0.050 |

Abbreviations: AP, anteroposterior; BMI, body mass index

^a^ We included patients hospitalized after out-of-hospital cardiac arrest and excluded age under 18, patients who transferred from other hospital, same as the study cohort.

Values are presented as number (%) or median (interquartile range). Categorical variable was tested by chi-square test, and continuous variables were calculated with the Mann-Whitney test. **p* < 0.05 is significant.
